# Persistent Post-COVID-19 Olfactory Dysfunction and Its Association with Autonomic Nervous System Function: A Case–Control Study

**DOI:** 10.3390/diseases13010004

**Published:** 2024-12-28

**Authors:** Lojine Ayoub, Abeer F. Almarzouki, Rajaa Al-Raddadi, Mohamed A. Bendary

**Affiliations:** 1 Department of Physiology, Faculty of Medicine, Rabigh Branch, King Abdulaziz University, Rabigh 21911, Saudi Arabia; 2 Department of Clinical Physiology, Faculty of Medicine, King Abdulaziz University, Jeddah 21589, Saudi Arabia; afmalmarzouki@kau.edu.sa (A.F.A.); mamohamad2@kau.edu.sa (M.A.B.); 3 Department of Community Medicine, Faculty of Medicine, King Abdulaziz University, Jeddah 21589, Saudi Arabia

**Keywords:** post-COVID-19, subjective and objective smell tests, autonomic dysfunction

## Abstract

Background: Following the Coronavirus Disease 2019 (COVID-19) pandemic, many patients have reported ongoing smell and taste issues. This study aims to investigate the prevalence of olfactory and gustatory dysfunction among patients with a history of COVID-19 and its association with autonomic dysfunction and disability. Patient and Methods: This case–control study included a COVID-19 group (n = 82) and a control group (n = 82). Olfactory dysfunction, including parosmia and taste problems, was explored using self-reports and the Quick Smell Identification Test (QSIT). The association between post-COVID-19 disability severity and taste and smell alterations was also analyzed. Moreover, autonomic function was evaluated using the Composite Autonomic Symptom Scale-31 (COMPASS-31) to assess the association between autonomic and olfactory dysfunction. Results: Significantly higher rates of ongoing smell (26.8%) and taste (14.6%) dysfunction were reported for the post-COVID-19 group compared to the control group. Post-COVID-19 patients reported 36.6 times more smell issues and 8.22 times more taste issues than controls. Parosmia scores were significantly worse in the post-COVID-19 group, while QSIT scores showed no significant difference between the groups. However, those with worse QSIT scores exhibited significantly more ongoing smell issues. No significant association was observed between disability and altered smell or taste. Higher secretomotor dysfunction scores were significantly associated with abnormal QSIT scores and worse parosmia scores; the other domains of the COMPASS-31 scale showed no significant associations. Conclusions: The findings indicated a potential link between autonomic and olfactory dysfunction. Further studies are needed to elucidate the mechanisms underlying persistent olfactory and autonomic dysfunction in post-COVID-19 patients.

## 1. Introduction

The prevalence of symptoms in patients with a history of Coronavirus Disease 2019 (COVID-19) infection ranges from 14.34% to 63.2% among those with a history of COVID-19 infection [1,2]. These symptoms might appear after recovery or persist following acute illness and may fluctuate or recur over time [3]. Common symptoms include tiredness, dyspnea, headache, and loss of smell, which can interfere with daily activities [3,4]. Studies found that COVID-19-vaccinated individuals were 29% less likely to develop post-COVID-19 symptoms compared to unvaccinated patients [5]. The risk was decreased further to 36% with two doses of vaccination compared to those with no vaccination [6]. One of the most common persistent symptoms of post-COVID-19 is olfactory dysfunction, a notable symptom of acute COVID-19 since the beginning of the pandemic [7,8].

Olfactory dysfunction among COVID-19 cases includes anosmia, hyposmia, and phantosmia. The latter refers to a complete loss or reduced sense of smell, as well as olfactory hallucinations in the absence of smell stimuli [9,10,11]. In addition, patients experience “parosmia”, which comprises distorted smell perception in the presence of smell stimuli [7,8,12]. It was reported that approximately 60.5% of patients with a history of COVID-19 infection experienced smell and taste issues [13]. Persistent olfactory dysfunction symptoms can lead to changes in eating habits, nutritional deficiencies, anxiety, depression, and diminished quality of life [14,15,16,17].

Olfactory function tests are mainly used to examine the individual’s sense of smell and diagnose olfactory dysfunction. Each type of olfactory function test has special characteristics and clinical application. Subjective smell tests depend on individuals’ self-reporting of the perception of odor. On the other hand, objective smell tests depend on a standard measurable reproducible criterion in evaluating smell function [18,19].

Olfactory dysfunction treatments such as olfactory training (OT), which involves daily exposure to various odors, have been shown to improve olfactory symptoms when performed early following COVID-19 infection, rather than at a later stage [20,21]. However, a deeper understanding of the mechanisms underlying COVID-19’s effect on the olfactory system is fundamental for developing targeted treatments to improve olfactory function in affected individuals. The factors linked to long-lasting post-COVID-19 olfactory dysfunction remain poorly understood [22]. Sex has been reported as a nonmodifiable risk factor for olfactory dysfunction, which is more common in women. Other risk factors include nasal allergy and current smoking [23,24]. Disability has been linked to persistent olfactory dysfunction in post-COVID-19 patients in many previous studies [25,26,27].

COVID-19 infection has also been found to affect autonomic nervous system (ANS) function [28]. Symptoms following COVID-19 infection may be caused by a disturbance in the ANS [4]. Autonomic dysfunction implies malfunction or excessive parasympathetic or sympathetic division activity with respect to the ANS. Common autonomic symptoms include orthostatic intolerance, impaired secretomotor and bladder function, and alterations in bowel function [29]. Common secretomotor symptoms in patients following COVID-19 infection are altered sweating and dryness of the eyes and mouth [30,31,32,33]. A study comparing a patient’s autonomic function before and after COVID-19 found that newly developed or aggravated sudomotor dysfunction symptoms were reported among all participants [30]. Between 67 and 95.1% of post-COVID-19 patients were reported to have moderate to severe autonomic dysfunction regardless of hospitalization status during acute COVID-19 illness [34,35], with autonomic dysfunction proposed as one of the underlying reasons for the persistent neurological symptoms of post-COVID-19 patients [34].

There are limited studies indicating a link between olfactory dysfunction and autonomic dysfunction in COVID-19 patients, which could be explained by shared or overlapping pathological mechanisms. One possible mechanism is neuroinflammation caused by the SARS-CoV-2 invasion of the brain via the olfactory pathway, which, in turn, affects both olfactory and autonomic system function [36]. Neuroinflammation contributes to the severe autonomic dysfunction associated with anosmia in COVID-19 patients [37].

The link between olfactory and autonomic dysfunction in COVID-19 survivors has not yet been firmly established. Establishing this link may open avenues for appropriate assessments and interventions in these patients.

Therefore, the principal aim of this study was to investigate the association between autonomic and olfactory dysfunction in patients with a history of laboratory-confirmed COVID-19 infection. Other objectives include identifying the predictors of persistent olfactory dysfunction in these patients and examining the association between persistent olfactory dysfunction and the degree of disability.

## 2. Patients and Methods

Ethical approval for this case–control study was obtained from The Unit of Biomedical Ethics Research Committee at King Abdulaziz University (KAU), Faculty of Medicine, on 18 April 2022. The approval number is 201-22.

Participants were recruited through an announcement that was distributed on notice boards within the Faculty of Medicine at KAU teaching hospital. The aim was to recruit a pool of participants with and without a history of COVID-19 infection, including university staff, students, and patients visiting the hospital. Interested participants contacted the research team. Participants were asked if they had a documented history of polymerase chain reaction (PCR) tests for COVID-19. Based on their responses, we assigned the participants to two groups:

1. ***The case group*** consisted of participants with a positive PCR history of COVID-19.
2. ***The control group*** comprised participants with no positive PCR history of COVID-19 and at least one negative PCR history of COVID-19.

The inclusion criteria were as follows: adult volunteers above 18 years, male and female individuals, and residents of Saudi Arabia regardless of nationality. We excluded participants with allergic rhinitis, current flu or cold symptoms, or those who were pregnant. For the autonomic function assessment, we additionally excluded participants with abnormally high baseline blood pressure (systolic blood pressure ≥ 140 mmHg and diastolic blood pressure ≥ 90 mmHg), those taking medications that affect the ANS (e.g., antihypertensives, beta blockers, calcium channel blockers, ACE inhibitors, alpha-1 blockers, antidepressants, antihistamines, and cholinesterase inhibitors), or those diagnosed with autonomic disorders.

This case–control study took place at the Clinical Physiology Department, Faculty of Medicine, KAU, Jeddah, Saudi Arabia. Patient recruitment lasted for 10 months from May 2022 to February 2023. The 164 participants who volunteered for this study signed a consent form and answered a questionnaire after receiving clear explanations regarding informed written consent and the aim and methods of the study. This was followed by anthropometric measurements and a smell evaluation test.

## 3. Methodology

Three different validated questionnaires were used. The first addressed acute and post-COVID-19 clinical assessment details, the second focused on olfactory perception, and the third was the Composite Autonomic Symptom Scale 31 (COMPASS-31). The olfactory section comprised inquiries designed to assess the subjective reporting of ongoing smell or taste problems, as well as parosmia.

COVID-19 infection was clinically assessed using modules 1 and 2 of the WHO global clinical platform for the COVID-19 Case Report Form (CRF) for COVID-19 Sequelae (“Post COVID-19 CRF”) [38]. Module 1 covers background demographic and clinical information on acute COVID-19 infection, including demographics, pre-existing conditions, details on acute COVID-19 information and diagnosis, and clinical management during the episode. Module 2 focuses on the period following acute COVID-19 infection and includes additional details for patients requiring further clinical evaluation. It is divided into six sections: hospital admission, reinfection, COVID-19 vaccination status, occupational status, functioning (WHO Assessment Schedule—WHO DAS 2.0), and the incidence of symptoms after acute COVID-19 illness, including altered smell and taste related to COVID-19.

Self-reported smell or taste issues following COVID-19 were evaluated using the WHO Module 2 post-COVID-19 symptom assessment, and they were categorized as either present at the time of the infection, at the time of the study, or not present (i.e., resolved or never experienced).

Patients were categorized according to the severity of acute COVID-19 disease following the WHO clinical classification grouping system. Mild cases referred to patients without hypoxia, pneumonia, or the need for oxygen. Moderate cases included patients who did not receive oxygen therapy but developed non-severe pneumonia, with SpO_2_ levels higher than 90% on room air. Severe cases involved patients receiving noninvasive oxygen therapy or those exhibiting clinical signs of severe pneumonia with blood oxygen levels less than 90% with respect to room air or a respiratory rate higher than 30 breaths per minute. In critical cases, patients received invasive ventilation and/or suffered from conditions such as respiratory distress syndrome (RDS), septicemia, multi-inflammatory syndrome, lung thromboembolism, coronary heart disease, or acute cerebrovascular accident linked to COVID-19 [38].

In addition, we used the WHO Disability Assessment Schedule (WHODAS) to classify post-COVID-19 patients according to their current level of disability. The WHODAS data were manually calculated using a simple scoring system. Total scores from items 1 to 12 were categorized into four levels of disability: none (zero points), mild (one to four points), moderate (five to nine points), or severe (ten to forty-eight points) [39].

The COMPASS-31 questionnaire was used to assess autonomic function. COMPASS-31 is a self-administered questionnaire for evaluating six domains of autonomic function: orthostatic intolerance, vasomotor, secretomotor, gastrointestinal, bladder, and pupillomotor domains [40,41,42]. The overall weighted scores for COMPASS-31 and its domains were calculated following the original guidelines [43]. COMPASS-31 measures of autonomic dysfunction were excluded in 10 participants who had abnormally high baseline blood pressure (5 per group).

The parosmia questionnaire was also used to assess qualitative olfactory function. It consisted of four questions, each with four possible answers; these were assigned the following scores: always (1), often (2), rarely (3), and never (4) [12]. We calculated the total parosmia score by summing up the points from all the answers. We then calculated Parosmia Score A using the following equation: (Total Score − 4)/12) × 100 [44]. Parosmia Score A was used to group the parosmia scores into three categories: low (<50%), suggesting more severe parosmia; moderate parosmia (50–84%); and high (≥85%), representing less severe or absent parosmia [44].

A standardized Quick Smell Identification Test (QSIT; Sensonics International, Haddon Heights, NJ, USA) was used as an objective screening tool to assess olfactory function in each individual. The QSIT (scored zero through three) was used to measure issues with smell. It consists of three scratch-and-sniff micro-encapsulated standardized scents presented on disposable tear-out cards. The patient was presented with five multiple-choice options for each odor, including “none/other” as one of the options. The first question assesses chocolate odor, the second banana odor, and the third smoke odor.

The QSIT was selected due to its disposable (tear-off card) format, which eliminates any potential for contamination or disease transmission from COVID-19 patients. The test is rapid (less than 1 min), noninvasive, and cost-effective [12]. The QSIT was validated by the University of Pennsylvania Smell Identification Test (UPSIT). The three odorants utilized in QSIT were validated, and they were accurately identified by our community [45]. The QSIT was also validated in previous studies conducted on COVID-19 patients [46,47].

The cut-off point for an abnormal QSIT score is ≤2. Therefore, a result is considered abnormal when individuals score 0, 1, or 2 on this test [12].

Anthropometric measurements were recorded in kilograms for each participant, weighed using an electronic scale with a height rod (electronic scale with height rod, FAZZINI srl, No. S7350HR, Milan, Italy) [48]. The waist circumference was measured to the nearest 0.1 cm in a standing position using stretch-resistant measuring tape, as recommended by the WHO [49]. The body mass index (BMI) was calculated by dividing weight in kilograms by height in square meters. We also classified the study population into the following four different categories according to BMI (kg/m^2^): underweight (below 18.5), normal (18.5 to below 25), overweight (25 to below 30), or obese (30 or above).

## 4. Statistical Analysis

The Statistical Package for the Social Sciences (SPSS program version 20, Chicago, IL, USA) was used for data analysis. Model residuals were confirmed as normally distributed using the Kolmogorov–Smirnov test and were visually evaluated using QQ plots. Associations with binary outcomes were tested using logistic regression modeling, with the odds ratio (OR) reported as contrasts. Continuous normally distributed variables were described and expressed as mean and standard deviation (SD) and analyzed using unpaired Student *t*-test. Categorical variables were described and expressed as frequencies and % (percentage) and analyzed using Chi-square (χ^2^) test. Statistical models included age, sex, smoking status, and QSIT scores as covariates, adjusting for confounding of these factors. For all comparisons, a *p*-value < 0.05 was considered to indicate a statistically significant difference.

## 5. Results

### 5.1. Group Characteristics

The studied groups consisted of 82 control participants and 82 post-COVID-19 patients (Table 1). Participants were, on average, 22.58 years of age (SD = 6.28) and ranged from 18 to 62 years. The post-COVID-19 and control groups were similar in age, sex, BMI, and the prevalence of smoking.

Characteristics in post-COVID-19 patients (n = 82) and control group (n = 82). Continuous normally distributed variables (age and BMI) are presented as mean (standard deviation (SD)) and analyzed using unpaired Student *t* test. Categorical variables (sex, BMI categories, and smoking) are presented as frequencies and % (percentage) and analyzed using Chi-square (χ^2^) test. There was no statistically significant difference observed between the post-COVID-19 group and the control group in terms of age, sex, BMI, and smoking. * indicates significant differences between groups (*, *p* < 0.05). BMI = body mass index. A BMI below 18.5 is underweight, a BMI more than or equal to 18.5 kg/m^2^ but less than 25 kg/m^2^ is a normal BMI, a BMI more than or equal to 25 kg/m^2^ but less than 30 kg/m^2^ is overweight, and a BMI more than or equal to 30 kg/m^2^ is obese.

### 5.2. Clinical History

Among the post-COVID-19 group, 98% (n = 80) had a mild infection, with 1% (n = 1) experiencing a severe infection and 1% (n = 1) experiencing a critical infection. One participant was admitted to the hospital, three were treated as outpatients, and all others self-treated their illness. In this group, data were, on average, collected 311 days (SD = 237) after infection. The control group had no known history of COVID-19 infection. Participants in the post-COVID-19 group were categorized into WHODAS disability groups as follows: none (18%, n = 15), mild (26%, n = 21), moderate (29%, n = 24), and severe (27%, n = 22). Additionally, a small number (4%, n = 3) experienced complications from respiratory infection. In total, 93% of participants in the control group and 95% in the post-COVID-19 group reported three vaccinations, with a small number reporting either two (6% and 4%) or four (1% and 1%) vaccinations. No participant had fewer than two or more than four vaccinations.

### 5.3. Influence of COVID-19 on Smell and Taste

The post-COVID-19 group recorded significantly more ongoing smell and taste issues and exhibited worse parosmia scores both in terms of raw scores and category of severity (Table 2). Associations were found between these outcomes and several predictors. Logistic regression models for categorical outcomes (self-reported smell and taste issues and abnormal QSIT scores) revealed significant differences between the post-COVID-19 and the control groups. Group differences were also found in the linear regression models for continuous outcomes (parosmia scores). These significant differences remained after adjusting for confounding effects of participant age, sex, and smoking status and including these factors as covariates. The influence of all of these predictors is shown in Table 3. Self-reported smell issues were also more prevalent in those with abnormal QSIT scores. Notably, the significant difference in self-reported smell and taste issues between groups remained when controlling for the nonsignificant effects of smoking on each outcome (Table 3). The proportion of both normal and abnormal QSIT scores by group did not significantly differ at the threshold between normal and abnormal scores. The vast majority (94%) of participants reported having had three vaccinations, followed by two (5%) or four (1%) vaccinations.

In contrast to both self-reported smell/taste issues and parosmia scores, where a significant difference between groups was revealed, there was no significant change in QSIT scores (Table 2). Parosmia scores were not significantly associated with QSIT (t (161) = 1.92, *p* = 0.057). Although a higher proportion of the participants with self-reported smell issues had abnormal QSIT scores (21%) compared to those without smell issues (11%), this difference was not significant (χ^2^ = 1.09, *p* = 0.295).

## 6. Predictors of Olfactory Dysfunction

Issues with smell following recovery from acute illness were reported as having been experienced but resolved by 30% of post-COVID-19 participants or not resolved by another 26%, with no reported issues in the remaining participants. Issues with taste were similarly reported as resolved by 26% and unresolved by 17% of participants. Those with worse QSIT scores were significantly more likely to report ongoing smell issues (Table 3). Notably, while a significantly higher proportion of individuals who reported smell issues had abnormal QSIT scores (78%, χ^2^ = 5.99, *p* = 0.014 *), 48.2% of those who did not report smell issues still showed objective impairments (as measured by the QSIT, a standardized olfactory function test).

**Figure 1:**
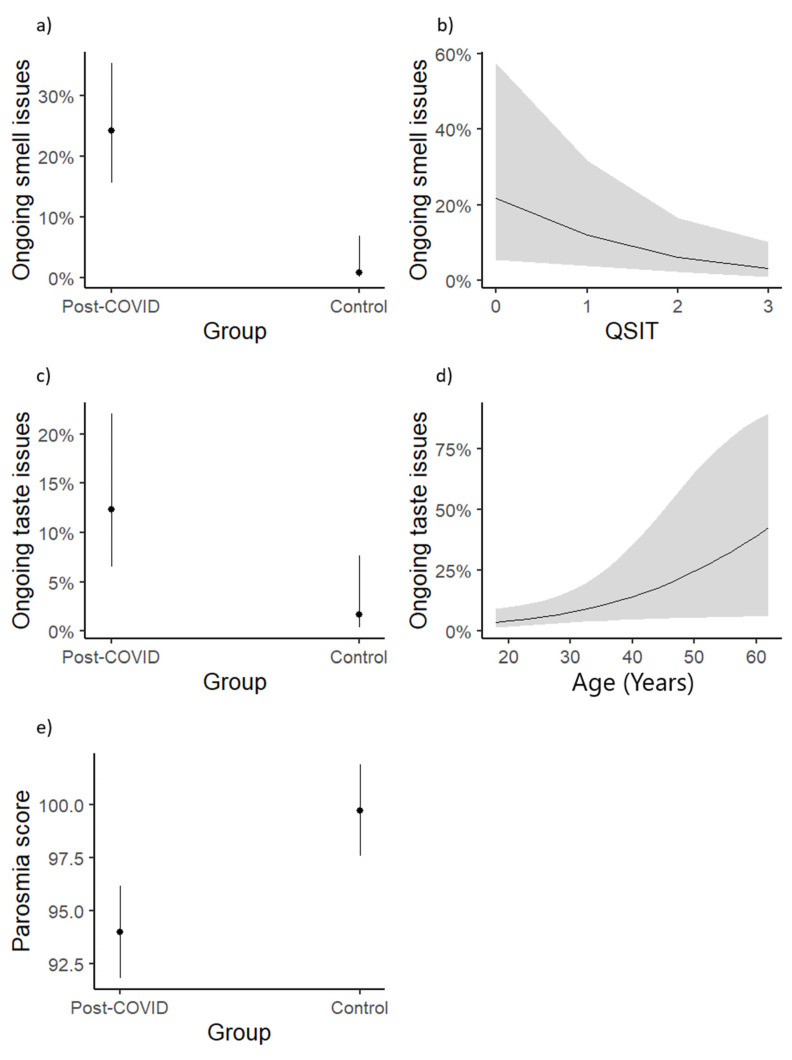
Significant influences on the prevalence of ongoing smell and taste issues and parosmia. Predictors of olfactory dysfunction in post-COVID-19 patients (n = 82) and control group (n = 82). Associations are shown between ongoing smell issues with group (**a**) and QSIT score (**b**), between ongoing taste issues with group (**c**) and age in years (**d**), and between parosmia with group (**e**). Parosmia Score equation: (Total Score − 4)/12) × 100. Error bars and shaded areas represent the 95% confidence interval around model estimates.. QSIT = Quick Smell Identification Test.

We used logistic regression modeling to identify group differences in ongoing smell (Figure 1a) or taste issues (Figure 1c) (one model for each outcome) and abnormal QSIT scores (Figure 1b) and to assess the associations between these outcomes and QSIT scores while adjusting for age, sex, and having been a smoker at or prior to the time of interview (Figure 1d). Similarly, linear regression was employed to test for associations of these outcomes with parosmia scores (Figure 1e).

Notably, the post-COVID-19 group experienced smell issues 36.6 times more relative to the control group when adjusting for other factors. In addition, they experienced taste issues 8.22 times more, even when adjusting for the significant effect of age on taste and the nonsignificant effect of smoking. While there were no group differences in QSIT scores, the post-COVID-19 group showed significantly worse parosmia scores (Table 3).

## 7. Association of Olfactory and Autonomic Dysfunction

**Figure 2:**
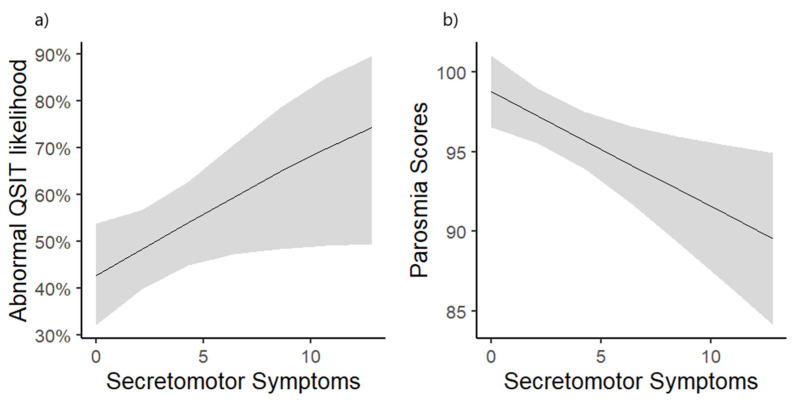
Association between higher secretomotor symptom scores as per Composite Autonomic Symptom Scale 31 (COMPASS-31) questionnaire item totals with more likely abnormal QSIT (scores of 2 or lower) (**a**) scores and worse parosmia scores (**b**). Logistic (abnormal QSIT score) and linear (parosmia score) regression model estimates were derived while adjusting for participant age, smoking status (past or present smoker vs. never), and duration since COVID-19 infection. Shaded areas represent the 95% confidence interval around model estimates. QSIT = Quick Smell Identification Test.

To test for a possible link between olfactory and autonomic dysfunction, we examined associations between normal vs. abnormal scores for QSIT and parosmia through scores for each of the six COMPASS-31 domains individually while controlling for age, having ever smoked, and duration since COVID-19 infection. Participants with higher weighted secretomotor dysfunction scores were significantly more likely to have abnormal QSIT scores (Z = 2.00, *p* = 0.046 *) (Figure 2a) and lower (worse) parosmia scores (Z = −2.74, *p* = 0.007) (Figure 2b).

## 8. Post-COVID-19 Disability

While 18% of participants showed no ongoing disability, others had mild (n = 26%), moderate (n = 29%), or severe (n = 27%) disability following infection. The degree of disability was not significantly related to duration since infection (F (3,78) = 0.25, *p* = 0.864).

**Figure 3:**
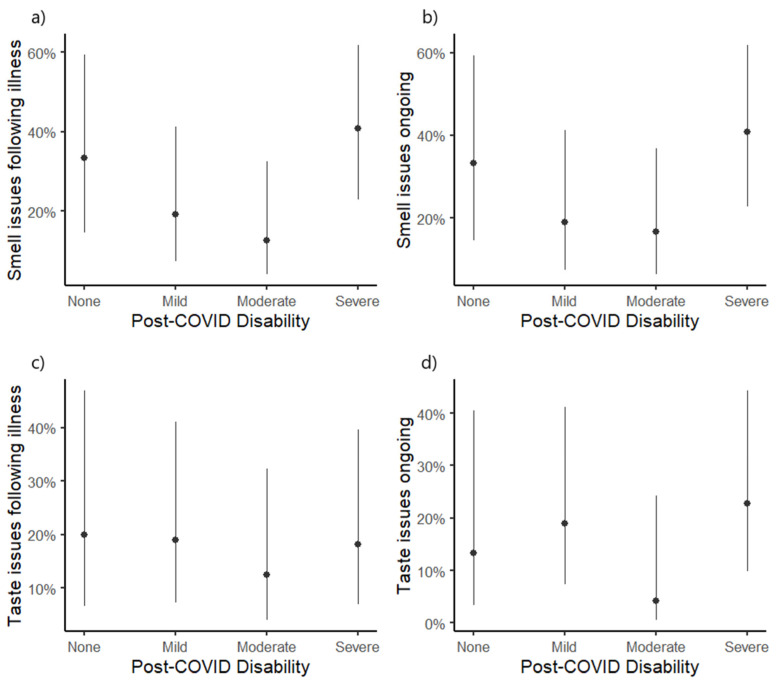
Association between post-COVID-19 disability groups (measured using the WHO Disability Assessment Schedule) and the presence of smell issues following acute illness (**a**), ongoing post-COVID-19 smell issues (**b**), taste issues following acute illness (**c**), and ongoing post-COVID-19 taste issues (**d**). Estimates show predicted probability as calculated using logistic regression modeling, with error bars depicting the 95% confidence interval around model estimates. The post-COVID disability group was not significantly associated with any of the four outcomes. Total simple scoring system of WHODAS scores categorize disability levels: none (0 points), mild (1–4 points), moderate (5–9 points), or severe (10–14 points).

There were no significant differences between disability groups in having experienced issues with smell either following acute illness (χ^2^ = 5.91, *p* = 0.116) (Figure 3a) or at the time of the interview (χ^2^ = 4.43, *p* = 0.218) (Figure 3b). Similarly, there were no differences in the prevalence of taste issues following acute illness (χ^2^ = 0.545, *p* = 0.919) (Figure 3c) or at the time of the interview (χ^2^ = 4.15, *p* = 0.246) (Figure 3d). These associations are shown in Figure 3.

Among the post-COVID-19 group, the prevalence of medium (13.4%) or more severe (2.4%) parosmia symptoms was low, with the vast majority (87.2%) showing perfect scores. The presence of medium or more severe symptoms did not vary by disability group (χ^2^ = 2.17, *p* = 0.538). Lastly, disability groups did not differ in terms of QSIT scores (F (3,78) = 0.04, *p* = 0.988) or rates of abnormal QSIT scores (χ^2^ = 0.38, *p* = 0.945).

## 9. Discussion

In the current study, we evaluated the long-term effects of COVID-19 on olfaction and its association with autonomic function and disability by comparing individuals who had a history of COVID-19 with a control group. Significantly higher rates of ongoing smell and taste issues were reported for the post-COVID-19 group compared to the control group, a difference that remained significant even after adjusting for age, sex, and smoking status. The rate of reported smell issues was 26.8%, which is consistent with previous studies in the region [9,10] and internationally [22]. Parosmia is considered a qualitative olfactory disorder, with parosmia prevalence rates in previous studies observed to vary between 40.8% and 70.9% among patients who had COVID-19 [11,20,50,51,52]. In our study, we found a significant difference in parosmia scores between the control and post-COVID-19 groups, with a parosmia rate of 15.9% among post-COVID-19 patients. These differences from previously reported studies could be caused by several variables, such as discrepancies in regions, evaluation methodology, and sample demographics.

In our study, parosmia scores were significantly worse in the post-COVID-19 group, while QSIT scores showed no significant difference. Unreported dysfunction can be identified through objective quantitative olfactory function tests such as the QSIT, which provides quantifiable, uniform measures of olfactory function [18]. However, a significant limitation of the QSIT is that it underestimates the presence of different types of olfactory dysfunction. The QSIT alone may underestimate the prevalence of COVID-related smell issues. This suggests adding complementary tests to strengthen the discrimination of COVID-related smell issues. Therefore, adding subjective qualitative olfactory function to objective olfactory function tests is needed, specifically those that assess parosmia. Subjective tests can be used when the focus is on individual experiences and for the detection of more qualitative olfactory disorders such as parosmia [18]. A high rate of smell issues was reported for our sample. However, no significant differences between the groups were found on the basis of the QSIT. This can be explained by the type of olfactory dysfunction that dominated our sample, which was indicated by the high rates of parosmia. This implies that QSIT is a useful preliminary tool that, however, should be supplemented with more comprehensive tests and clinical evaluations for detecting and managing quantitative and qualitative olfactory dysfunction in post-COVID-19 patients [19,53,54].

Our results reveal that patients with worse QSIT scores are significantly more likely to report ongoing smell issues, which aligns with previous studies [47]. For example, in a study in Saudi Arabia, a significant correlation was also found between objective smell tests and the subjective participants’ reports of anosmia or hyposmia [51]. However, unexpectedly, we found that almost half of the patients who reported normal olfactory function still had an abnormal QSIT. This suggests that individuals may not be aware of their olfactory dysfunction, which could lead to the prevalence of olfactory dysfunction in post-COVID-19 patients being underreported in studies where only subjective olfactory assessments are used. This again emphasizes the need to combine both subjective and objective smell tests in evaluating olfactory function in post-COVID-19 patients [18]. Although a considerable proportion of post-COVID-19 participants reported ongoing disability, ranging from mild to severe, there was no significant association found between the degree of disability and the prevalence of olfactory dysfunction. The degree of disability was not significantly related to the time period since acute COVID-19 infection. This finding mostly indicates that, in cases where olfactory issues were more prevalent, it remains inconclusive whether there is a direct association with overall disability experienced by post-COVID-19 individuals.

On the other hand, examination of the olfactory and taste dysfunctions following COVID-19 infection has revealed a positive correlation between disability and reduced olfactory scores [27]. Though, in our study, there were no statistical differences detected, post-COVID-19 patients with moderate disability levels reported having fewer smell and taste issues when compared to those in the mild group. One possible explanation could be due to the subjective variability in self-reporting of symptoms in patients. Another explanation for the difference in our results compared to other studies may be that our sample primarily comprises relatively young individuals without pre-existing comorbidities. This, in turn, could affect disability outcomes.

Olfactory function is influenced by many factors, including demographic, clinical, genetic, and lifestyle factors. While age commonly affects smelling capabilities [55,56], it was not found to be a significant predictor in our study. This could be due to the young average age of participants (mean of 22.58 ± 6.28 years). Olfactory function declines with normal aging [57]. Therefore, it is possible that younger participants have different baseline olfactory scores than older participants. This will also contribute to the difference in the prevalence and degree of olfactory dysfunction reported in post-COVID-19 patients. Sex was also not a significant predictor of olfactory function, despite conflicting findings in the literature [23,58]. Similarly, allergies and smoking, while often considered risk factors for olfactory dysfunction, were not significant predictors [24]. On the other hand, in line with our study, previous studies have shown that post-COVID-19 infection and parosmia are significant predictors of olfactory dysfunction [54,59,60]. Overall, multiple factors influence the persistence and recovery of olfactory dysfunction in post-COVID-19 patients.

Hyposmia, or lack of smell, may be a sign of pure autonomic failure [61]. A similar study highlighted the significance of having a fully functional autonomic nerve system in the process of identifying odors, for which individuals diagnosed with pure autonomic failure exhibited diminished ability [62]. Studies on COVID-19 have found an association between olfactory symptoms and neurological symptoms [36,37,60,63]. Orthostatic intolerance was more frequent among COVID-19 patients with anosmia (63.6%) than those without (37.5%) [37]. One study on post-COVID-19 patients found a significant association between brain fog and parosmia, while another found an association between chemosensory dysfunction and neuropsychiatric symptoms [52,64]. Moreover, the effects of treatments in reducing olfactory dysfunction symptoms in post-COVID-19 patients, such as stellate ganglion block, are thought to be autonomically mediated [60,63].

Our COMPASS-31 questionnaire results showed that the secretomotor domain is associated with abnormal QSIT results and worse parosmia scores in post-COVID-19 patients. These results are consistent with previous studies examining the relationship between olfactory dysfunction and autonomic symptoms. In their study, Cremaschi et al. also used the COMPASS-31 questionnaire, although they focused on two questions out of four questions from the secretomotor domain to assess xerophthalmia and xerostomia instead of reporting results across all six domains [37]. However, their study results showed that two symptoms corresponding to the secretomotor domain were significantly more prevalent in post-COVID-19 patients with olfactory dysfunction. These results further support the possibility of a shared pathological mechanism between the olfactory and autonomic nervous systems in post-COVID-19 patients.

The pathophysiology of autonomic and olfactory dysfunction in COVID-19 patients may involve numerous pathways. Angiotensin-converting enzyme 2 (ACE2) receptors are the principal target of the SARS-CoV-2 virus [65]. These receptors are mainly expressed in the respiratory and olfactory epithelium, in addition to being found in extrapulmonary sites [37]. SARS-CoV-2 binds to ACE2 receptors and invades the olfactory mucosa in association with transmembrane protease serine type 2 (TMPRSS2), which mediates S-protein cleavage [66]. Sustentacular cells support the olfactory neurons and are high in TMPRSS2 expression [67]. This, in turn, leads to its higher susceptibility to infection, resulting in disruption of olfactory signal transduction pathways and symptoms of olfactory dysfunction [68,69]. Multiple hypotheses explain the development of persistent olfactory dysfunction symptoms following COVID-19. Studies indicated that SARS-CoV-2 may persist in human tissues long after the acute infection. The presence of the virus in the tissues may contribute to persistent symptoms in those patients. Autopsies have identified extensive viral replication and distribution in multiple tissues [70]. In patients with persistent smell loss, persistent viral presence and inflammation in the olfactory mucosa have been detected [71]. These results indicate that continued inflammation and viral persistence may lead to prolonged or relapsing COVID-19 symptoms, including olfactory dysfunction [72]. Another hypothesis that explains the development of persistent olfactory dysfunction is associated with significant damage to the olfactory epithelium [66,68]. The damage increases the resistance to cerebrospinal fluid outflow, causing congestion within the glymphatic system and the accumulation of toxins in the brain [36]. Moreover, SARS-CoV-2 infection leads to endothelial dysfunction and the triggering of a considerable coagulation cascade. This can potentially result in multiple organ dysfunction, particularly of the autonomic nervous system function via the disruption of the angiopoietin/Tie axis and a consequent inflammatory response [73]. Furthermore, the virus can access the systemic circulation and cross the blood–brain barrier [28,36]. Multiple brain regions, such as the choroid plexus and olfactory bulb, express the ACE-2 receptors, which makes them highly susceptible for SARS-CoV-2 infection. The ability of SAR-CoV-2 to infect and replicate in the nervous system cells is known as SARS-CoV-2 neurotropism [74]. This also results in both olfactory and autonomic dysfunctions in post-COVID-19 patients. Moreover, SARS-CoV-2–ACE2 interaction may result in sustained immune activation [37]. It is possible that ACE2 antibodies amplify pre-existing immunological responses, thereby exacerbating dysfunctions in post-COVID-19 patients.

There are several limitations of this case–control study. First, as with any case–control study, the study’s design is limited to establishing causality. Second, sample recruitment was convenience-based and carried out via university-related networks. This may result in selection bias, as the participants might not represent the general population. In addition, most of the participants were young and university-affiliated, which further decreases diversity of the sample. This homogeneity limits the generalizability and the external validity of the findings to other age groups. In future studies, attempts should be carried out using broader and more randomized recruitment methods to reduce selection bias and to create a more diverse and representative sample with a wider range of ages and varying degree of disability, therefore, improving the generalizability and external validity of the results and exploring the possibility of COVID-19 affecting individuals differently in patients with varying degrees of disability. Future studies should also consider using both subjective and objective olfactory tests to ensure a more comprehensive olfactory assessment and that the accurate prevalence of post-COVID-19 olfactory dysfunction is not underestimated [75]. Finally, we classified the participants into case and control groups based on the PCR COVID-19 test. For more precise case–control classification, future studies should use the serological testing of nucleocapsid (N protein) antibodies [76]. These antibodies are present in patients previously infected with COVID-19, even if they are asymptomatic. Therefore, this could also help identify controls who were never infected.

## 10. Conclusions

The results of this study provide new insights into the broader impact of COVID-19. We propose a potential shared pathophysiological mechanism based on the significant correlation between persistent olfactory dysfunction, specifically parosmia, and autonomic dysfunction. The connection between olfactory and autonomic dysfunction, including the molecular mechanisms underlying the interaction between the associated systems, should be determined through further research. We also conclude that both subjective and objective olfactory tests are needed for accurately diagnosing olfactory dysfunction. Further research is needed to explore possible targeted treatments for post-COVID-19 patients experiencing persistent concurrent symptoms of olfactory and autonomic dysfunction.

## Figures and Tables

**Table 1:** Characteristics of control group and post-COVID-19 patients’ group in context of age, sex, BMI, and smoking.

	Control	Post-COVID-19	Group Difference
**Sample Size**	82	82	
**Sex**			
Male—frequency (%)	24 (29.3)	26 (31.7)	χ^2^ = 0.03, *p* = 0.865
Female—frequency (%)	58 (70.7)	56 (68.3)	
**Age—Mean (SD)**	22.85 (6.90)	22.30 (5.61)	t (162) = −0.56, *p* = 0.577
**BMI—Mean (SD)**	24.38 (6.18)	24.28 (6.23)	t (162) = −0.1, *p* = 0.918
**BMI Categories—** **frequency (%)**			χ^2^ = 0.54, *p* = 0.910
*Underweight*	11 (13.4)	14 (17.1)	
*Normal*	40 (48.8)	37 (45.1)	
*Overweight*	15 (18.3)	14 (17.1)	
*Obese*	16 (19.5)	17 (20.7)	
**Smoking Status—** **frequency (%)**			χ^2^ = 3.54, *p* = 0.171
*Never*	64 (78.0)	65 (79.3)	
*Former*	1 (1.2)	5 (6.1)	
*Current*	17 (20.7)	12 (14.6)	

**Table 2:** Prevalence of smell and taste issues in control group and post-COVID-19 patients’ group.

	Control	Post-COVID-19	Group Difference
**Sample size**	82	82	
**Ongoing smell issues—frequency (%)**	1 (1.2)	22 (26.8)	χ^2^ = 20.23, *p* < 0.001 *
**Ongoing taste issues—frequency (%)**	2 (2.4)	12 (14.6)	χ^2^ = 6.33, *p* = 0.012 *
**Parosmia score—mean (SD)**	99.80 (1.84)	93.90 (14.05)	t (162) = −3.77, *p* < 0.001 *
**Parosmia Subcategories—frequency (%)**			χ^2^ = 11.29, *p* = 0.004 *
Less severe	81 (98.8)	69 (84.1)	
Medium	1 (1.2)	11 (13.4)	
More severe	0 (0.0)	2 (2.4)	
**QSIT Score—frequency (%)**			χ^2^ = 2.91, *p* = 0.406
*Zero*	1 (1.2)	3 (3.7)	
*One*	6 (7.3)	11 (13.4)	
*Two*	35 (42.7)	30 (36.6)	
*Three*	40 (48.8)	38 (46.3)	
QSIT 2 or lower—frequency (%)	42 (51.2)	44 (53.7)	χ^2^ = 0.02, *p* = 0.876

Significance testing of variables between control (n = 82) and post-COVID-19 (n = 82) groups. The continuous normally distributed variable (parosmia score) is presented as mean (SD) and analyzed using unpaired Student *t*-test. Categorical variables (ongoing smell issues, ongoing taste issues, and QSIT score) are presented as frequencies and % (percentage) and analyzed using Chi-square (χ^2^) test. Parosmia Score equation: (Total Score − 4)/12) × 100. Parosmia scores: (<50%) more severe parosmia; (50–84%) moderate parosmia; and (≥85%), representing less severe or absent parosmia. The post-COVID-19 group reported significantly higher ongoing smell and taste issues and exhibited worse parosmia scores both in terms of raw scores and category of severity. * Significant differences between groups (*, *p* < 0.05). QSIT = Quick Smell Identification Test.

**Table 3:** Predictors of olfactory dysfunction comparing post-COVID-19 patients to control group.

		OR [95% CI]	Estimate (SE)	Significance
Smell issues	QSIT	0.49 [0.27, 0.89]	−0.71 (0.30)	Z = −2.35, *p* = 0.020 *
	Age	1.06 [0.99, 1.14]	0.06 (0.04)	Z = 1.69, *p* = 0.090
	Sex (Male)	1.40 [0.46, 4.32]	0.34 (0.57)	Z = 0.59, *p* = 0.550
	Smoker	0.51 [0.12, 2.09]	−0.68 (0.73)	Z = −0.94, *p* = 0.350
	Group (Post-COVID-19)	36.58 [4.16, 321.29]	3.60 (1.11)	Z = 3.25, *p* = 0.000 *
Taste issues	QSIT	0.53 [0.26, 1.03]	−0.63 (0.34)	Z = −1.87, *p* = 0.060
	Age	1.07 [1.01, 1.14]	0.07 (0.03)	Z = 2.04, *p* = 0.040 *
	Sex (Male)	0.58 [0.13, 2.53]	−0.54 (0.75)	Z = −0.72, *p* = 0.470
	Smoker	2.31 [0.57, 9.35]	0.84 (0.71)	Z = 1.18, *p* = 0.240
	Group (Post-COVID-19)	8.22 [1.59, 42.66]	2.11 (0.84)	Z = 2.51, *p* = 0.010 *
QSIT < 3	Age	0.98 [0.93, 1.03]	−0.02 (0.03)	Z = −0.90, *p* = 0.370
	Sex (Male)	1.26 [0.63, 2.53]	0.23 (0.35)	Z = 0.66, *p* = 0.510
	Smoker	0.79 [0.36, 1.72]	−0.24 (0.40)	Z = −0.59, *p* = 0.550
	Group (Post-COVID-19)	1.08 [0.58, 2.00]	0.08 (0.31)	Z = 0.25, *p* = 0.810
Parosmia	QSIT	n/a	1.66 (1.04)	t (158) = 1.61, *p* = 0.110
	Age	n/a	−0.16 (0.13)	t (158) = −1.30, *p* = 0.190
	Sex (Male)	n/a	0.96 (1.75)	t (158) = 0.55, *p* = 0.580
	Smoker	n/a	1.76 (1.96)	t (158) = 0.90, *p* = 0.370
	Group (Post-COVID-19)	n/a	−5.76 (1.56)	t (158) = −3.69, *p* < 0.001 *

Regression models comparing post-COVID-19 patients (n = 82) to control group (n = 82). Logistic regression models are used for categorical variables (smell issues, taste issues, and QSIT) and linear regression model for continues variable (parosmia). Model predictors included QSIT results (0–3), participant age, sex, smoking status (past or present smoker vs. never), and group (control or post-COVID-19). For logistic regression models, odds ratios (ORs) and their 95% confidence intervals (95% CI) are included. Model estimates and their standard error (SE) showed the effect of each predictor. OR and estimates refer to the relative difference for males, relative to females, and the post-COVID-19 group, relative to controls, on outcomes. * Significant influence of predictors on outcomes (*, *p* < 0.05). Reported smell issues were significantly associated with lower QSIT scores and more prevalent with worse parosmia score in the post-COVID-19 group. QSIT = Quick Smell Identification Test.

## Data Availability

The original contributions presented in this study are included in the article material. Further inquiries can be directed to the corresponding author.
